# Pin-Site Myiasis Caused by Screwworm Fly in Nonhealed Wound, Colombia

**DOI:** 10.3201/eid2502.181053

**Published:** 2019-02

**Authors:** Wilmer E. Villamil-Gómez, Jaime A. Cardona-Ospina, Juan Sebastián Prado-Ojeda, Hugo Hernández-Prado, Mauricio Figueroa, Pedro N. Causil-Morales, Keirim Pérez-Reyes, Leidy A. Palechor-Ocampo, Alfonso J. Rodríguez-Morales

**Affiliations:** Hospital Universitario de Sincelejo, Sincelejo, Colombia; Universidad del Atlántico, Barranquilla, Colombia (W.E. Villamil-Gómez, J.S. Prado-Ojeda. P.N. Causil-Morales);; Universidad Tecnológica de Pereira, Pereira, Colombia (J.A. Cardona-Ospina, L.A. Palechor-Ocampo, A.J. Rodríguez-Morales);; Clínica Santa María, Sincelejo (H. Hernández-Prado, M. Figueroa, K. Pérez-Reyes);; Universidad Privada Franz Tamayo/UNIFRANZ, Cochabamba, Bolivia (A.J. Rodríguez-Morales

**Keywords:** myiasis, pin site, *Cochliomyia hominivorax*, complication, chronic infection, screwworm fly, Colombia, parasites, external metal stabilizers, surgical wound, wound care, pin-site myiasis

## Abstract

Pin-site myiasis is an underreported complication of surgical interventions. We present a case of myiasis caused by the New World screwworm fly (*Cochliomyia hominivorax*) in a pin site of a chronic nonhealed wound 12 years after the intervention. This infection apparently was the result of poor perfusion of the leg.

Pin-site myiasis, a surgical complication reported since 2005 (*1*), is an infection with insect larvae in wounds after use of metal stabilizers to treat bone fractures. Although it is considered rare, its real incidence is unknown, probably because of underreporting. However, pin-site myiasis remains an important complication of surgical interventions when it occurs, particularly in patients with risk factors such as medical comorbidities, poor care of pin site, and advanced age (*2*). Although pin-site myiasis is nonfatal if diagnosed and treated, the tissue damage and secondary bacterial infection are known to have evolved in animals to septicemia and even death (*3*). For these reasons, it is important to keep this complication in the clinical spectrum of postoperative occurring conditions, especially in susceptible populations. We report a case of pin-site myiasis in an elderly patient with a chronic nonhealed wound.

A 77-year-old man with a history of hypertension who had tibial osteosynthesis in 2006 was admitted to the emergency service of Clínica Santa María, a local private hospital in Sincelejo, Sucre, Colombia, in May 2018. Four days earlier, he had noticed the presence of larvae as well as ulceration, bone exposure, and osteosynthesis material in a nonhealed wound in his left leg at the site where a pin had been inserted as part of his care 12 years earlier. The surgical wound had never healed after the intervention, and he was caring for the wound with homemade measures under poor hygiene. The patient denied exposure to pets, livestock, or wildlife. At his admission, he was afebrile and nonseptic, and vital signs were within reference levels. Examination of the leg revealed absence of pedial pulse, an ulcer of 8 cm in diameter, thickness of the skin and soft tissues surrounding the wound, bone exposure and osteosynthesis material, and larvae (Figure, panel A). A radiograph of the leg showed a bone callus and a functional posteriorly blocked pin, which was retired. We performed ultrasonography of arterial vessels, which showed atheromatosis of the popliteal artery with very low flow. After microbiological sampling of the secretions in the wound, we started intravenous cefazoline (1 g every 6 h) and washed the ulcer. The microbiological cultures were positive for oxacilin-resistant *Staphylococcus aureus*. The patient received vancomycin, with posterior negative cultures.

**Figure:**
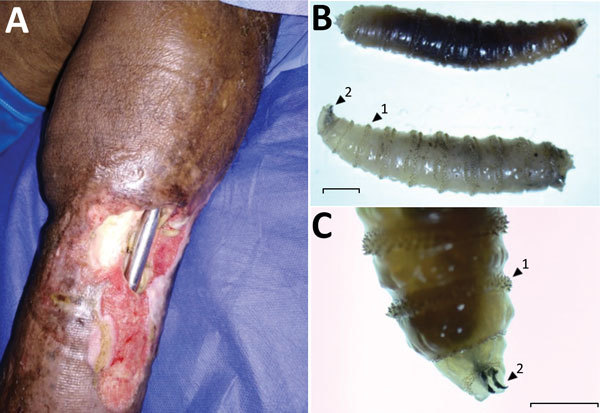
Pin-site myiasis in a 77-year-old man 12 years after tibial osteosynthesis, Colombia. A) Open wound in the man’s left leg, showing multiple insect larvae. B, C) *Cochliomyia hominivorax* screwworm fly larvae extracted from the wound. Arrow 1 indicates the spinose bands; note the spines arranged in 4 rows that separate each segment. Arrow 2 indicates its mouthhooks. Scale bars indicate 2 mm (B) and 1 mm (C).

We removed a total of 100 larvae from the wound and identified them, using published methods (*4*), as larvae of *Cochliomyia hominivorax*, the New World screwworm fly (Figure, panel B); the larvae have well-differentiated mouthhooks and 12 segments separated by spinose bands with spines arranged in 4 rows and an opened posterior spiracle. The identification of the larvae is based primarily on the presence or absence of internal breathing tubes (Figure, panel C). The life cycle of *C. hominivorax* flies is ≈21 days in warm climates, such as this patient’s area of residence, and slightly longer in cooler climates. The adult female mates only once and lays her elongated white eggs along the edges of wounds on warm-blooded animals.

After 4 weeks of antimicrobial therapy and daily debridement and irrigation, the wound appeared to be healing without evidence of bacterial or parasitic infection. Monthly follow-up for up to 6 months is expected.

Other authors have previously reported pin-site infestation with maggots; we found a total of 7 cases since 2005 (*1*,*2*,*5*–*7*). We did not find reports of a case in which the infestation complicates a chronic nonhealed surgical wound in the pin site 12 years after intervention. This patient had medical comorbidities and poor care of the pin site, as did previously reported case-patients (*8*). Ultrasonographic evaluation of the leg revealed poor perfusion, which probably affected the healing of the wound.

This case highlights the role of myiasis as a complication of surgical wounds (*2*,*6*), especially in pin sites. Appropriate debridement, washing, and antimicrobial treatment for bacteria and ectoparasites should help to prevent evolution of the infection to osteomyelitis and sepsis.
